# The Provision of Public Abattoirs

**Published:** 1907-06-08

**Authors:** 


### THE PROVISION OF PUBLIC ABATTOIRS.

Since the revelations made last year of the methods employed by certain American packing houses the popular interest then aroused on the subject of good meat has once more subsided into indifference. At no time has any great heed been paid by the public to the much more important matter of the provision in this country of healthy meat, and of healthy meat only. It is not therefore surprising to find that local authorities have not been quick to avail themselves of their powers of providing public abattoirs. And in this respect the larger towns of this country compare very unfavourably with those of our Continental neighbours. From time to time people become alarmed at the vast possibilities there are of purchasing and consuming unsound food, but with the subsidence of the scare there is a disappearance of all effort to see that the supervision of our food supplies is improved.

It may be said at once that the system prevailing in this country of slaughtering animals under conditions in which adequate inspection is impossible, as in private slaughter-houses, is one of serious public danger. It is not possible with the staff available to inspect the carcasses of the greater number of animals intended for human food. The difficulties of detecting disease in the fragments of meat exposed in a butcher's shop are well known, and the evidence of some of the most dangerous diseases, such as tuberculosis, has, for the purposes of practical inspection, virtually disappeared with the offal. It is recognised that proper protection can be secured only by the systematic inspection of each animal at the slaughter-house, and that this is impossible where there exist innumerable private slaughter-houses in which slaughtering is carried out at irregular and intermittent periods. The only person present to judge whether the animal be fit for human food in these circumstances is the butcher who is directly interested in the sale of the flesh.

Among other recommendations of the Third Royal Commission on Tuberculosis was one in these terms:—

"1. Where there is a public slaughter-house the Local Authority shall have power to prohibit slaughtering in any other place.

"2. The Local Authority shall have power to require that the meat of animals slaughtered elsewhere than in a public slaughter-house and brought into a district for sale be taken to some place where it may be inspected.

"3. Where a public slaughter-house has been established inspectors shall inspect all carcasses immediately after slaughter and stamp all such as are sound."

Were these recommendations given legislative effect it would be possible to enjoy a sense of confidence in the soundness of our meat supplies that at present is wholly lacking.

The provision of public abattoirs, in fact, is the first—and, so far as our home supplies are concerned, the most essential—step in the development of an efficient system of inspection of our animal food. The utmost we can expect for the present, however, are the sporadic attempts of the more enlightened municipalities to provide private slaughter-houses and so protect their citizens from the grave dangers arising from the present inadequacy of inspection.

